# Comparative analysis of the vaginal microbiome of pregnant women with either *Trichomonas vaginalis* or *Chlamydia trachomatis*

**DOI:** 10.1371/journal.pone.0225545

**Published:** 2019-12-12

**Authors:** Simon Chengo Masha, Collins Owuor, Joyce Mwongeli Ngoi, Piet Cools, Eduard J. Sanders, Mario Vaneechoutte, Tania Crucitti, Etienne P. de Villiers

**Affiliations:** 1 KEMRI-Wellcome Trust Research Programme, Centre for Geographic Medicine Research-Coast, Kilifi, Kenya; 2 Laboratory for Bacteriology Research, Faculty of Medicine and Health Sciences, Ghent University, Ghent, Belgium; 3 Department of Biological Sciences, Pwani University, Kilifi, Kenya; 4 Nuffield Department of Clinical Medicine, University of Oxford, Oxford, United Kingdom; 5 HIV/STI Reference Laboratory, Department of Clinical Sciences, Institute of Tropical Medicine, Nationalestraat, Antwerp, Belgium; 6 Centre for Tropical Medicine and Global Health, Nuffield Department of Medicine Research Building, University of Oxford, Oxford, United Kingdom; 7 Department of Public Health, Pwani University, Kilifi, Kenya; Fred Hutchinson Cancer Research Center, UNITED STATES

## Abstract

**Background:**

Although the significance of the human vaginal microbiome for health and disease is increasingly acknowledged, there is paucity of data on the differences in the composition of the vaginal microbiome upon infection with different sexually transmitted pathogens.

**Method:**

The composition of the vaginal bacterial community of women with *Trichomonas vaginalis* (TV, N = 18) was compared to that of women with *Chlamydia trachomatis* (CT, N = 14), and to that of controls (N = 21) (women negative for TV, CT and bacterial vaginosis). The vaginal bacterial composition was determined using high throughput sequencing with the Ion 16S metagenomics kit of the variable regions 2, 4 and 8 of the bacterial 16S ribosomal RNA gene from the vaginal swab DNA extract of the women. QIIME and R package “Phyloseq” were used to assess the α- and β-diversity and absolute abundance of the 16S rRNA gene per sample in the three groups. Differences in taxa at various levels were determined using the independent T-test.

**Results:**

A total of 545 operational taxonomic units (OTUs) were identified in all the three groups of which 488 occurred in all three groups (core OTUs). Bacterial α-diversity, by both Simpson’s and Shannon’s indices, was significantly higher, (p = 0.056) and (p = 0.001) respectively, among women with either TV or CT than among controls (mean α-diversity TV-infected > CT-infected > Controls). At the genus level, women infected with TV had a significantly (p < 0.01) higher abundance of *Parvimonas* and *Prevotella* species compared to both controls and CT-infected women, whereas women infected with CT had a significantly (p < 0.05) higher abundance of *Anaerococcus*, *Collinsella*, *Corynebacterium* and *Dialister*.

**Conclusion:**

The vaginal microbiomes of TV and CT-infected women were markedly different from each other and from women without TV and CT. Future studies should determine whether the altered microbiomes are merely markers of disease, or whether they actively contribute to the pathology of the two genital infections.

## Introduction

The human body is estimated to host approximately 4 * 10^13^ bacterial cells, roughly equaling the number of human cells [1]. Bacteria represent the major part of microbiomes on the human body and have been shown to play a significant role in human health, affecting development of the immune system, nutrition and weight, among other physiological aspects [2]. Instead of distinct microbial species being either beneficial or harmful to human health, changes in the global balance of the microbiome might play a more crucial function [3]. Therefore, it is important to understand how the bacterial communities may vary under different circumstances.

Genital infections with either *Trichomonas vaginalis* (TV) or *Chlamydia trachomatis* (CT) were estimated to affect over a quarter of a billion women worldwide in 2012 [4]. These two pathogens invade the vaginal microbiome despite its resilience upon disturbance, e.g. by causing low pH [5, 6]. A vaginal microbiome associated with vaginal health is typically characterized by the predominance of one or few species of lactobacilli [7]. *Lactobacillus crispatus* and *L*. *iners* are the most prevalent vaginal lactobacilli in women of reproductive age, [7–9] and *L*. *crispatus* plays a major role in maintaining the vaginal environment in equilibrium, whereas *L*. *iners* has been associated with both eubiotic vaginal microbiomes as well as dysbiotic vaginal microbiomes associated with bacterial vaginosis (BV) or sexually transmitted infections (STIs) [9].

*T*. *vaginalis* has been associated with a vaginal pH ≥ 4.5 [10]. The more alkaline vaginal pH associated with TV would potentially lead to invasion of other pathogens, whilst further decreasing the concentration of lactobacilli which thrive in an acidic environment. Martin *et al*. [11] suggested that TV infection was associated with *Mycoplasma hominis* and Candidatus *Mycoplasma girerdii*, a species almost exclusively found in women infected with TV [12].

Vaginal dysbiosis has been associated with an increased risk of STIs, including *C*. *trachomatis* [13]. Imbalances in the vaginal microbiome, such as bacterial vaginosis, are important during pregnancy, because they have been associated with an increased risk of post-abortion infection [14], both early and late miscarriage [15, 16], preterm premature rupture of membranes [17], and preterm birth [18]. Preterm birth complications are of unique importance as they are estimated to be responsible for 14% of the world’s annual neonatal deaths [19].

Here, we determined the vaginal microbiome profiles of three groups of pregnant women (TV-infected, CT-infected, and controls, *i*.*e*. women with none of the two infections) attending antenatal care at Kilifi, County Hospital, Kenya, and we compare our results with previous findings.

## Materials and methods

We obtained ethical approval from the Kenya Medical Research Institute Scientific and Ethics Review Unit (#3022). Written informed consent was obtained from all the participants.

This report utilized repository samples from a study of 350 pregnant Kenyan women, as previously described [20]. Briefly, from July until September 2015, women attending the antenatal care clinic of Kilifi County Hospital, Kenya, were recruited into a cross-sectional study. The main aim of that study was to describe the prevalence and the predictors of curable STIs among pregnant women [20]. Women were eligible if they met the following criteria: age 18–45 years, gestation ≥ 14 weeks, being residents of the Kilifi Health and Demographic Surveillance area, willingness to undergo free STI and BV screening procedures and willing to give written informed consent.

For the above-described study, a nurse collected vaginal secretions from the vaginal introitus using three sterile cotton swabs. The first vaginal swab was inoculated at the clinic in the upper-chamber of an InPouch system (BioMed Diagnostics, White City, Oregon), a highly specific and sensitive device containing a fluid medium supporting the growth of *Trichomonas vaginalis* (TV) and allowing microscopic observation of TV. The inoculated InPouch was transferred to the laboratory within 15 minutes for direct microscopy of the upper chamber, after which it was merged with the lower chamber and incubated at 37°C ± 1°C. Daily microscopic observation (at both ×10 and ×40 magnification, for six fields) of the InPouch system was performed by qualified technicians. Samples with motile trichomonads within 5 days of culture were considered positive for TV.

The second swab was used for bacterial vaginosis (BV) diagnosis using the scoring system described by Nugent [21].

The third swab was placed in a sterile labelled 2 ml Eppendorf tube and the swab shaft was broken by bending the shaft against the neck of the Eppendorf tube. The bottom portion of the swab with the specimen was transported to the laboratory where it was immediately stored dry at -80°C for molecular studies of the vaginal microbiome, and the PCR for TV. No transport or freezing medium was added. *Chlamydia trachomatis* (CT) and *Neisseria gonorrhoeae* detection was performed on fresh first catch urine, using the GeneXpert® CT/NG Assay (Cepheid, Sunnyvale, California), according to the manufacturer’s instructions.

### DNA extraction

Before DNA extraction, the frozen swabs were thawed at room temperature for 30 minutes. Extraction was performed using the QIAamp DNA Mini Kit (Qiagen, Hilden, Germany) according to manufacturer’s instructions and 160 μl of eluted DNA was transferred to Eppendorf tubes and frozen at -80°C until molecular analysis was performed.

Further, PCR for *T*. *vaginalis* targeting the actin gene, using primers, previously described [12] yielding a fragment of 1100 bp were carried out on the ABI Veriti thermocycler platform (ThermoFisher Scientific, Waltham, Massachusetts). The primers were synthesized by Eurogentec, Liège, Belgium. The PCR was carried out with primers Tv8S (5′-TCT GGA ATG GCT GAA GAA GAC G-3′) and Tv9R (5′-CAG GGT ACA TCG TAT TGG TC-3′), with the following thermocycling conditions: 5 min at 95°C, 40 cycles of 30 s at 95°C, 30 s at 55°C and 3 min at 72°C, followed by 7 min at 72°C. Amplified fragments were visualized under UV light after agarose gel electrophoresis and EthBr staining.

Specimens for this study are derived from the stored swabs. Due to financial and logistic constraints, we could process only a subset of the 350 vaginal swabs from the main study. Vaginal swabs from 53 pregnant women were divided into three groups for analysis: first group was of 18 vaginal swabs from women TV PCR-positive, second group was of 14 vaginal swabs from women PCR-positive for CT in urine. The third group or control group consisted of 21 vaginal swabs from women PCR-negative for both TV and CT in urine. No matching of women was performed while selecting the control group. The socio-demographics of the 21 women whose swabs were selected as controls were not different from the 251 not selected as controls (Table A in S1 File). All the vaginal swabs selected for this analysis were BV negative by Nugent score.

### Bacterial 16S ribosomal RNA (rRNA) gene PCR and sequencing

The Ion 16S Metagenomics^TM^ Kit (ThermoFisher Scientific, Waltham, MA) primer set V2-4-8 was used to amplify the hypervariable regions of the 16S rRNA gene from bacteria for all the samples and from a positive control (DNA from Microbial Mock Community A (HM-278D –BEI)), according to the kit instructions. The amplicons were then purified using the AMPure magnetic bead-based purification system (Beckman Coulter, Porterville, CA) and quantified using the Qubit® 2.0 Fluorometer and the Qubit dsDNA HS Assay kit (ThermoFisher Scientific).

Sequencing libraries were synthesized using the Ion Plus^™^ Fragment Library Kit (ThermoFisher Scientific) to ligate barcoded adapters to the amplicons. For quality control, each barcoded library was assessed in order to determine proper ligation of the adapters by checking the size distribution and concentration of libraries. The Agilent Bioanalyzer (Agilent, Santa Clara, CA) was used for size distribution, while the library concentration was determined using the Ion Library TaqMan® Quantitation Kit (ThermoFisher Scientific) according to the kit instructions. The barcoded libraries were pooled in equimolar amounts with 25 libraries and one positive control (Microbial Mock Community A) pooled into one batch. Templating of the pooled library was done using the Ion PGM^™^ Hi-Q^™^ OT2 Kit on the OneTouch2^™^ system according to the manufacturer’s recommendations (ThermoFisher Scientific). The templated library was then enriched for template-positive Ion Sphere particles on the ion one touch ES system followed by 400-bp sequencing on the Ion Torrent PGM (ThermoFisher Scientific) using the Ion PGM^™^ Hi-Q^™^ Sequencing Kit and a 318 v2 chip (ThermoFisher Scientific).

### Data processing and statistical analysis

#### Identification of bacterial taxa in vaginal samples

After sequencing, the individual sequence reads were filtered by the PGM software to remove low quality and polyclonal sequences. Sequences matching the PGM 3′ adaptor were automatically trimmed. All PGM quality-approved, trimmed and filtered data were exported as sff files and further visualized and trimmed on quality in CLC Genomics Workbench version 9.5.3. Quality trimmed reads were exported as fasta files and processed using QIIME version 1.9.1 [22]. Sequences with a length between 200 and 250 bp, mean sequence quality score > 25 were retained.

Presence of homopolymers of > 6 bp, and sequences with mismatched primers were omitted. In order to calculate downstream diversity measures (α- and β-diversity indices, Unifrac analysis), 16S rRNA Operational Taxonomic Units (OTUs) were defined at ≥ 97% sequence homology using the open reference picking pipeline and taxonomy assignment workflow of QIIME and the Greengenes version 13.8 reference dataset [23].

#### α-diversity analysis

The α-diversity (diversity within samples) was evaluated using Simpson’s and Shannon’s indices. Samples were clustered into the three groups, i.e. CT-infected, TV-infected, and controls, and median diversity indices per group were compared using Analysis of variance (ANOVA). Pair-wise comparisons of the relative abundance of genera were computed using done by t-test.

β-diversity analysis was used to evaluate whether there was dissimilarity in variation between the three groups of women. Analysis of dissimilarity between groups was performed by Bray-Curtis (non-phylogeny based method that only takes the relative abundance of OTUs into account) and visualized through principal coordinates analysis (PCoA). The analysis of similarity (Anosim) test showed that the vaginal microbiome diversity within each group was not significantly different between the groups.

Non-metric multidimensional scaling (NMDS) analysis was applied to the dataset. PCoA was used to model the variation between samples based on the three groups.

The 16S rRNA gene sequences reported in this study have been submitted to the European Nucleotide Archive (ENA) under accession number PRJEB25935. Low abundant OTUs were excluded from subsequent analysis, i.e. only those OTUs were included that had a relative abundance of > 0.01 (assigned reads/total number of reads) in at least one sample. Data-mining and statistical analysis was done in Calypso version 8.54, available at (http://bioinfo.qimr.edu.au/calypso/), including visualization of the taxonomic information [24].

Chi-square was used to compare the socio-demographic and behavioral characteristics when comparing the groups of women on STATA version 13.1 (Stata Corp, College Station, Texas).

## Results

Two of the women with a TV infection were co-infected with HIV but both were not on anti-HIV therapy at the time of sample collection as HIV had just been diagnosed. There were no significant differences in the socio-demographic and behavioral characteristics of the three groups of women (Table 1). Finally, the socio demographic, behavioral, and clinical characteristics of 21 women whose swabs were selected as controls were not significantly different from the 251 women not selected as controls (Table A in S1 File).

**Table 1:** Socio-demographic, behavioral, and clinical characteristics of pregnant women (N = 53), attending antenatal care at Kilifi County Hospital, Kenya.

Characteristic	Controls (%) N = 21	TV cases (%) N = 18	CT cases (%) N = 14	χ2 P-value
**Age group (Years)**				
18–24	28.57	38.89	64.29	
≥ 25	71.43	61.11	35.71	0.106
**Religion**				
Christian	66.67	72.22	78.57	
Muslim	9.52	11.11	21.43	
Other/None	23.81	16.67	0.0	0.360
**Education**				
None	19.05	22.22	21.43	
Primary	61.90	61.11	57.14	
Secondary/Tertiary	19.05	16.67	21.43	0.996
**Parity**				
0	19.05	27.78	21.43	
1–2	42.86	44.44	57.14	
3+	38.10	27.78	21.43	0.814
**Gestational age (weeks)**				
14–27	66.67	55.56	53.85	
≥ 28	33.33	44.44	46.15	0.692
**Number of lifetime sex partners**				
≤ 2	95.24	77.78	64.29	
≥3	4.76	22.22	35.71	0.065
**HIV**				
Positive	0.0	11.11	0.0	
Negative	100.0	88.89	100.0	0.133
**Nugent scores**				
Normal (0–3)	95.24	55.56	85.71	
Intermediate (4–6)	4.76	44.44	14.29	**0.008**

**Fig 1:**
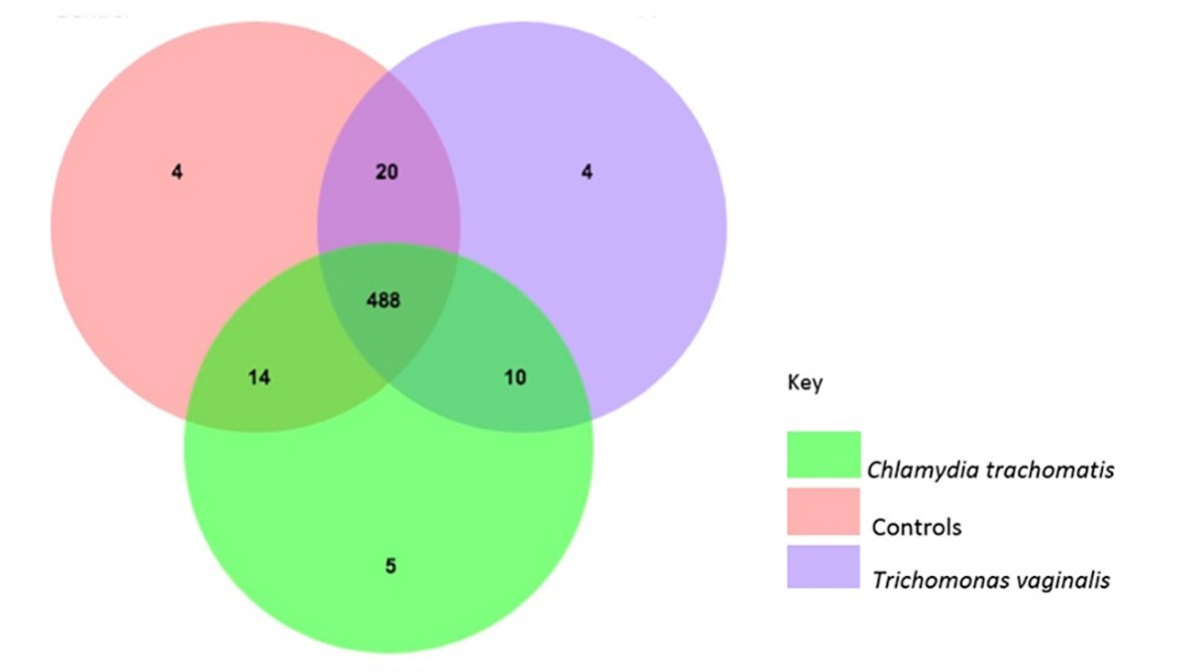
Venn diagram of the dominant OTU’s for each group. Numbers indicate the number of operational taxonomic units OTU’s.

To quantify the community composition of the three study groups of pregnant women, amplicons were clustered in operational taxonomic units (OTUs) using UCLUST [16] with a sequence similarity threshold of 97%. After quality filtering and chimeric checking, a total of 7232969 reads were assigned to 545 OTUs. A total of 488 OTUs were considered as the core OTUs since they occurred in all three groups. Controls and TV-infected women had 4 unique OTUs each, while CT-infected women had 5 unique OTUs (Fig 1).

**Fig 2:**
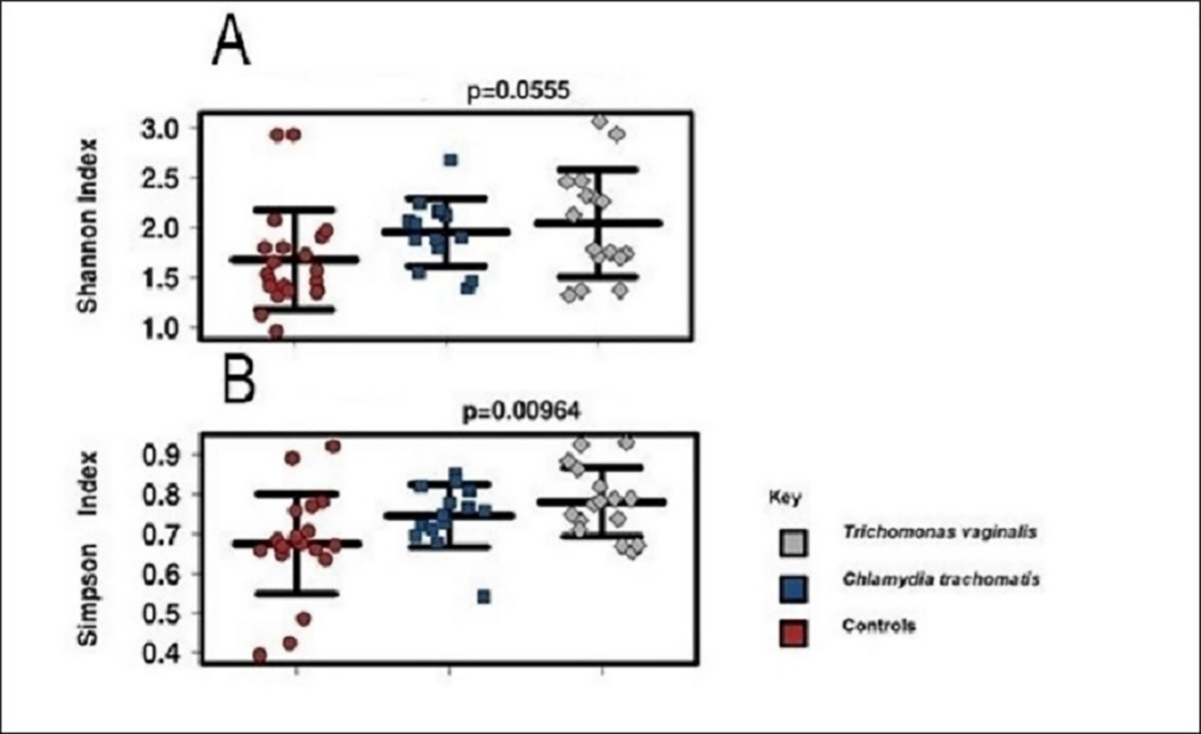
Bacterial α-diversity among the different groups of pregnant women in Kilifi, Kenya. Dot plots represent (A) Shannon index and (B) Simpson index. Statistical analysis was performed using ANOVA. Lines inside dot plots represent mean ± standard error.

Bacterial α-diversity was significantly higher among pregnant women with either TV or CT compared to controls, with the mean α-diversity of TV-infected > than that of CT-infected, that of CT-infected > than mean α-diversity of controls (Fig 2).

**Fig 3:**
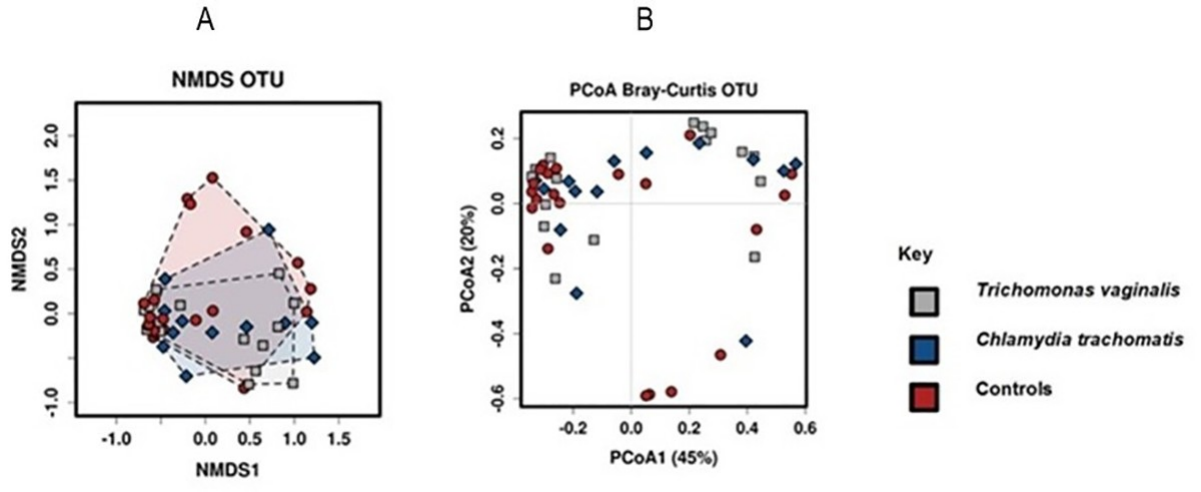
β-diversity of vaginal microbiomes among the different groups of pregnant women. β-diversity of vaginal microbiomes among pregnant women with *T*. *vaginalis* (n = 18), *C*. *trachomatis* (n = 14) and the control group (n = 21). (A) Non-metric multidimensional scaling (NMDS) plot of the vaginal bacteria of the three groups. Points represent the vaginal microbiomes of individual women at all taxonomic levels; colors indicate the infection status. (B) Principle coordinates analysis (PCoA) based on Bray Curtis metrics on Operational Taxonomic Units (OTUs).

The centroids, or average center, were not significantly different for the three groups (Fig 3).

**Fig 4:**
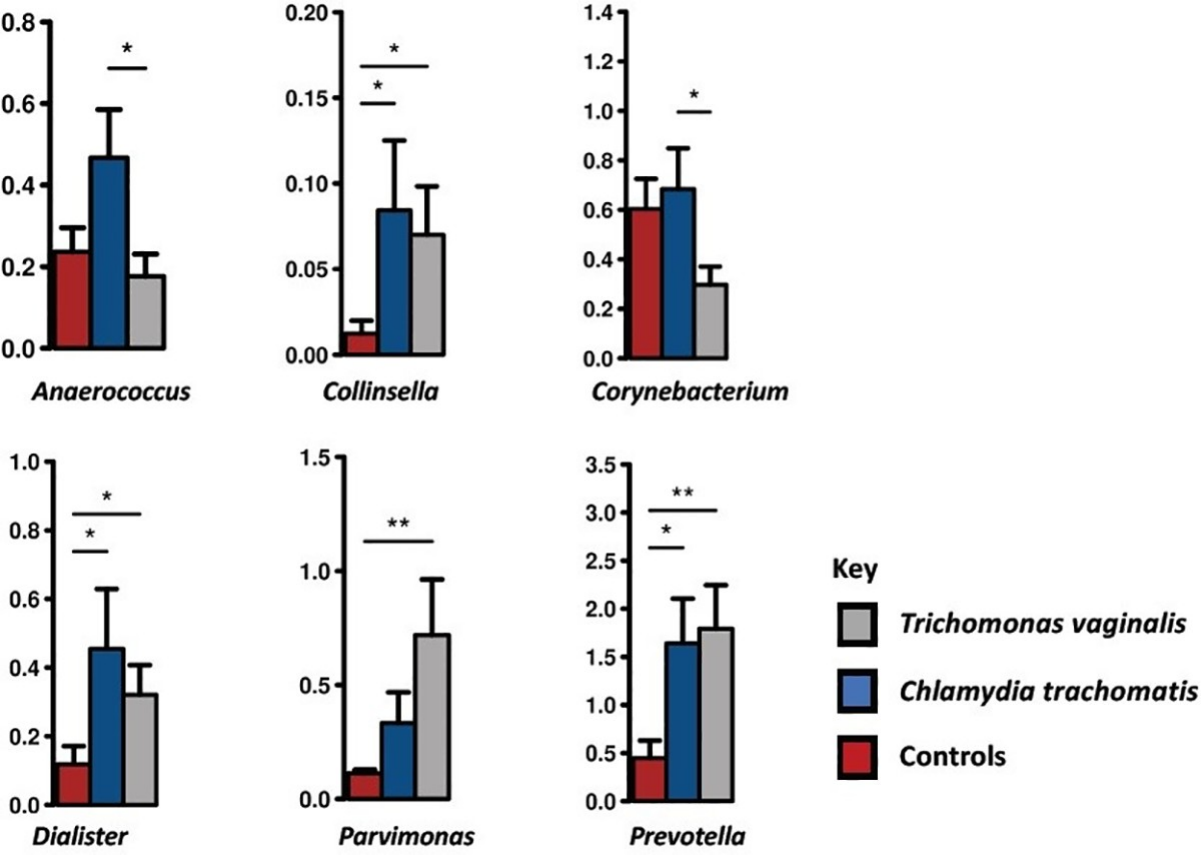
Bar graphs showing relative abundance of genera. The bar graphs are of genera that were significantly different among the three groups. Women with CT and TV had more Collinsella, Dialister and Prevotella than controls. Women with CT had more Anaerococcus and Corynebacterium than women with TV. Women with TV have more Parvimonas than controls. Pair-wise comparisons are done by t-test and annotated as *; p < 0.05, **; p < 0.01. Standard error is depicted by error bars.

Taxonomic similarities between each of the three groups were assessed by comparing the bacterial abundance in the three groups at various taxonomic levels. At the genus level, women infected with TV had a significantly (p < 0.01) higher abundance of *Parvimonas* and *Prevotella* compared to controls and to CT-infected women. Women infected with CT had in addition to abundant *Chlamydia*, a significantly (p < 0.05) higher abundance of *Anaerococcus*, *Collinsella*, *Corynebacterium* and *Dialister* (Fig 4).

At family level, the abundance of the *Lactobacillaceae* was evident for all three groups and not significantly different between groups (Figure A in S1 File).

We further performed analysis based on Nugent scores irrespective of genital infection status. We compared women with a normal Nugent score (0–3) (N = 42) with those with an intermediate Nugent score (4–6) (N = 11). Bacterial α-diversity was higher among women with intermediate Nugent scores as compared to those with normal Nugent scores (Figure B in S1 File). At family level, the abundance of *Aerococcaceae*, *Alcaligenaceae*, *Clostridiaceae*, *Coriobacteriaceae*, *Fusobacteriaceae*, *Gemellaceae*, *Lachnospiraceae*, *Lactobacillaceae*, *Leptotrichiaceae*, *Prevotellaceae* and *Tissierellaceae* was significantly different between the two groups (Figure C in S1 File)

## Discussion

This molecular epidemiological study, using high-throughput sequencing of the 16S rRNA gene of vaginal microbiomes intended to assess whether vaginal microbiomes differ among three groups of pregnant women: one group with genital infections with *Chlamydia trachomatis* (CT) (n = 14), one group with *Trichomonas vaginalis* (TV) infection (n = 18) and one control group, comprising (n = 21) women without CT and TV infection and without bacterial vaginosis (BV). We demonstrated increased α-diversity during infection with both TV and CT as compared to women not infected with either of both infectious agents. Since both indices used to determine the α-diversity (Simpson’s and Shannon’s) take into account the abundance and evenness of the OTUs present, this result suggests that infection with either TV or CT is associated with an increase in species diversity.

In a previous study [25], TV infection was associated with vaginal microbiomes consisting of low proportions of lactobacilli and high proportions of *Mycoplasma*, *Parvimonas*, *Sneathia* and other anaerobes. Martin *et al*. [11] similarly identified *Parvimonas* and *Prevotella* as significantly associated with TV infection. Also in our study, women infected with TV had significantly (p < 0.01) higher abundance of *Parvimonas* and *Prevotella* (Fig 4) as compared to controls. *Prevotella* has been shown to be elevated in women with BV [26]. Whether *Parvimonas* plays an active role in the pathology of TV or is rather a markers of infection with TV is not clear.

Several genera were more abundant among samples of women with CT included *Collinsella* and *Dialister* compared to controls and *Anaerococcus* and *Corynebacterium* compared to TV (Fig 4). We could not confirm the finding of Tamarelle *et al*. [27], who reported that infection with CT was associated with vaginal microbiomes dominated by *Lactobacillus iners* and largely lacking other *Lactobacillus* species respectively with a wide array of strict and facultative anaerobes.

The clinical relevance of genera associated with CT in our study varies. *Anaerococcus* has been isolated in cases of urinary tract infections [28]. Bacteria of the genus *Collinsella*, are not really known to be associated with vaginal dysbiosis. They are mainly found in the gut and their abundance in the gut of patients with rheumatoid arthritis correlates strongly with increased production of the proinflammatory cytokine IL-17A [29]. Bacteria of the genus *Corynebacterium* are increasingly being recognized as causing opportunistic infections in patients who are immunocompromised, have prosthetic devices, or have been in hospitals/nursing homes for long-term periods of time [30]. While *Dialister* species have been implicated in oral diseases [31], and recently *Dialister* has been identified as a microbial marker of disease activity in Spondyloarthritis. However, the importance of *Dialister* species in vaginal microbiome remains unknown [32].

Our study had a few limitations; first, we did not perform a biochemical analysis to exclude that the participants were on antibiotics. Antibiotics used for various treatments might also interfere with a healthy bacterial equilibrium in the vaginal microbiome, specifically causing a decrease in the prevalence of commensal *Lactobacillus* spp. However, we collected data using a questionnaire on antibiotic use and none of the participants declared to be on antibiotics. Second, no internal control was added during the DNA extraction process, so inefficient genome extraction may have occurred, although a genomic DNA from Microbial Mock Community A (HM-278D –BEI) was used as a positive control during amplification. Third, we only knew the status of a couple of STIs (CT, NG, TV, HIV, and *Treponema pallidum*), while other STIs not analyzed may have an effect on the microbiome as well. Fourth, during selection of the controls we did not match with number of sexual partners which potentially may have impacted on the likelihood of exposure to various microbes. However, the difference in number of sexual partners between the three groups was not statistically significant. Finally, our sample size was limited due to financial constraints.

## Conclusion

*Colinsella*, *Dialister* and *Prevotella* were significantly increased in CT- and TV-infected women compared to controls. *Anaerococcus* and *Corynebacterium* were most abundant in CT-infected women and significantly more abundant when compared to TV-infected women, and finally *Parvimonas* and *Prevotella* were most abundant in TV-infected women and significantly increased compared to controls. Future research should focus on the functional importance of the various vaginal bacteria. Longitudinal studies of vaginal microbiome and STIs are critical in order to determine causality, including its direction, and temporality.

## Supporting information

S1 File **Table A.** Socio-demographic and behavioral characteristics of women selected as controls and women not selected as controls, attending antenatal care at Kilifi County Hospital, Kenya.
**Figure A. Bar graph showing relative abundance of vaginal bacteria at family level for the three groups**. Bar graphs of taxa that are significantly different annotated as *: p<0.05, **: p<0.01, ***: p<0.001 for pair-wise comparisons by t-test for the three groups of women.
**Figure B. Bacterial α-diversity using Simpson and Shannon indices of women based on Nugent scores**. Legend. Intermediate Nugent score; 4–6 (n = 11), Normal Nugent score; (0–3) (n = 42).
**Figure C. Bar graph showing relative abundance at family level based on Nugent scores.** The bar chart display taxa that are significantly different taxa based on an Anova analysis Only taxa that are significantly different with a p-value < 1 are shown A Pair-wise comparisons is then done by t-test and annotated as *: p<0.05, **: p<0.01, ***: p<0.001 Standard error is depicted by error bars. Intermediate Nugent score: 4–6 (n = 11), Normal Nugent score: 0–3 (n = 42).
(DOCX)

## References

[1] Sender R, Fuchs S, Milo R. Revised Estimates for the Number of Human and Bacteria Cells in the Body. PLoS biology. 2016;14(8):e1002533 Epub 2016/08/20. 10.1371/journal.pbio.1002533 27541692 PMC4991899

[2] Gill SR, Pop M, DeBoy RT, Eckburg PB, Turnbaugh PJ, Samuel BS, et al Metagenomic Analysis of the Human Distal Gut Microbiome. Science (New York, NY). 2006;312(5778):1355–9. 10.1126/science.1124234 PMC3027896. 16741115 PMC3027896

[3] Human Microbiome Project C. Structure, function and diversity of the healthy human microbiome. Nature. 2012;486(7402):207–14. Epub 2012/06/16. 10.1038/nature11234 22699609 PMC3564958

[4] Newman L, Rowley J, Vander Hoorn S, Wijesooriya NS, Unemo M, Low N, et al Global Estimates of the Prevalence and Incidence of Four Curable Sexually Transmitted Infections in 2012 Based on Systematic Review and Global Reporting. PloS one. 2015;10(12):e0143304 Epub 2015/12/10. 10.1371/journal.pone.0143304 26646541 PMC4672879

[5] Alakomi HL, Skytta E, Saarela M, Mattila-Sandholm T, Latva-Kala K, Helander IM. Lactic acid permeabilizes gram-negative bacteria by disrupting the outer membrane. Applied and environmental microbiology. 2000;66(5):2001–5. Epub 2000/05/02. 10.1128/aem.66.5.2001-2005.2000 10788373 PMC101446

[6] Kaewsrichan J, Peeyananjarassri K, Kongprasertkit J. Selection and identification of anaerobic lactobacilli producing inhibitory compounds against vaginal pathogens. FEMS Immunology & Medical Microbiology. 2006;48(1):75–83. 10.1111/j.1574-695X.2006.00124.x 16965354

[7] Verhelst R, Verstraelen H, Claeys G, Verschraegen G, Van Simaey L, De Ganck C, et al Comparison between Gram stain and culture for the characterization of vaginal microflora: Definition of a distinct grade that resembles grade I microflora and revised categorization of grade I microflora. BMC microbiology. 2005;5(1):61 10.1186/1471-2180-5-61 16225680 PMC1266370

[8] Antonio MA, Hawes SE, Hillier SL. The identification of vaginal Lactobacillus species and the demographic and microbiologic characteristics of women colonized by these species. The Journal of infectious diseases. 1999;180(6):1950–6. Epub 1999/11/24. 10.1086/315109 . 10558952

[9] Vaneechoutte M. Lactobacillus iners, the unusual suspect. Research in microbiology. 2017;168(9–10):826–36. 10.1016/j.resmic.2017.09.003 . 28951208

[10] Bell C, Hough E, Smith A, Greene L. Targeted screening for Trichomonas vaginalis in women, a pH-based approach. International journal of STD & AIDS. 2007;18(6):402–3. Epub 2007/07/05. 10.1258/095646207781024892 . 17609030

[11] Martin DH, Zozaya M, Lillis RA, Myers L, Nsuami MJ, Ferris MJ. Unique Vaginal Microbiota That Includes an Unknown Mycoplasma-Like Organism Is Associated With Trichomonas vaginalis Infection. Journal of Infectious Diseases. 2013;207(12):1922–31. 10.1093/infdis/jit100 23482642 PMC3654749

[12] Masha SC, Cools P, Descheemaeker P, Reynders M, Sanders EJ, Vaneechoutte M. Urogenital pathogens, associated with Trichomonas vaginalis, among pregnant women in Kilifi, Kenya: a nested case-control study. BMC Infect Dis. 2018;18(1):549 10.1186/s12879-018-3455-4 30400890 PMC6219184

[13] Bautista CT, Wurapa EK, Sateren WB, Morris SM, Hollingsworth BP, Sanchez JL. Association of Bacterial Vaginosis With Chlamydia and Gonorrhea Among Women in the U.S. Army. American journal of preventive medicine. 2017;52(5):632–9. Epub 2016/11/07. 10.1016/j.amepre.2016.09.016 . 27816380

[14] Larsson PG, Platz-Christensen JJ, Dalaker K, Eriksson K, Fahraeus L, Irminger K, et al Treatment with 2% clindamycin vaginal cream prior to first trimester surgical abortion to reduce signs of postoperative infection: a prospective, double-blinded, placebo-controlled, multicenter study. Acta obstetricia et gynecologica Scandinavica. 2000;79(5):390–6. Epub 2000/06/01. . 10830767

[15] Ralph SG, Rutherford AJ, Wilson JD. Influence of bacterial vaginosis on conception and miscarriage in the first trimester: cohort study. Bmj. 1999;319(7204):220–3. Epub 1999/07/23. 10.1136/bmj.319.7204.220 10417083 PMC28171

[16] Llahi-Camp JM, Rai R, Ison C, Regan L, Taylor-Robinson D. Association of bacterial vaginosis with a history of second trimester miscarriage. Hum Reprod. 1996;11(7):1575–8. Epub 1996/07/01. 10.1093/oxfordjournals.humrep.a019440 . 8671507

[17] Parry S, Strauss JF 3rd. Premature rupture of the fetal membranes. N Engl J Med. 1998;338(10):663–70. Epub 1998/03/05. 10.1056/NEJM199803053381006 . 9486996

[18] Leitich H, Bodner-Adler B, Brunbauer M, Kaider A, Egarter C, Husslein P. Bacterial vaginosis as a risk factor for preterm delivery: a meta-analysis. American journal of obstetrics and gynecology. 2003;189 10.1067/mob.2003.339 12861153

[19] Liu L, Johnson HL, Cousens S, Perin J, Scott S, Lawn JE, et al Global, regional, and national causes of child mortality: an updated systematic analysis for 2010 with time trends since 2000. Lancet. 2012;379(9832):2151–61. Epub 2012/05/15. 10.1016/S0140-6736(12)60560-1 . 22579125

[20] Masha SC, Wahome E, Vaneechoutte M, Cools P, Crucitti T, Sanders EJ. High prevalence of curable sexually transmitted infections among pregnant women in a rural county hospital in Kilifi, Kenya. PloS one. 2017;12(3):e0175166 10.1371/journal.pone.0175166 . 28362869 PMC5375155

[21] Nugent RP, Krohn MA, Hillier SL. Reliability of diagnosing bacterial vaginosis is improved by a standardized method of gram stain interpretation. Journal of clinical microbiology. 1991;29(2):297–301. 1706728 10.1128/jcm.29.2.297-301.1991 PMC269757

[22] Caporaso JG, Kuczynski J, Stombaugh J, Bittinger K, Bushman FD, Costello EK, et al QIIME allows analysis of high-throughput community sequencing data. Nature methods. 2010;7(5):335–6. Epub 2010/04/13. 10.1038/nmeth.f.303 20383131 PMC3156573

[23] DeSantis TZ, Hugenholtz P, Larsen N, Rojas M, Brodie EL, Keller K, et al Greengenes, a chimera-checked 16S rRNA gene database and workbench compatible with ARB. Applied and environmental microbiology. 2006;72(7):5069–72. 10.1128/AEM.03006-05 16820507 PMC1489311

[24] Zakrzewski M, Proietti C, Ellis JJ, Hasan S, Brion MJ, Berger B, et al Calypso: a user-friendly web-server for mining and visualizing microbiome-environment interactions. Bioinformatics (Oxford, England). 2017;33(5):782–3. Epub 2016/12/28. 10.1093/bioinformatics/btw725 28025202 PMC5408814

[25] Brotman RM, Bradford LL, Conrad M, Gajer P, Ault K, Peralta L, et al Association between Trichomonas vaginalis and vaginal bacterial community composition among reproductive-age women. Sex Transm Dis. 2012;39(10):807–12. Epub 2012/09/26. 10.1097/OLQ.0b013e3182631c79 23007708 PMC3458234

[26] Srinivasan S, Fredricks DN. The human vaginal bacterial biota and bacterial vaginosis. Interdisciplinary perspectives on infectious diseases. 2008;2008:750479 Epub 2008/01/01. 10.1155/2008/750479 19282975 PMC2648628

[27] Tamarelle J, de Barbeyrac B, Le Hen I, Thiebaut A, Bebear C, Ravel J, et al Vaginal microbiota composition and association with prevalent Chlamydia trachomatis infection: a cross-sectional study of young women attending a STI clinic in France. Sexually transmitted infections. 2018 Epub 2018/01/24. 10.1136/sextrans-2017-053346 . 29358524

[28] Domann E, Hong G, Imirzalioglu C, Turschner S, Kuhle J, Watzel C, et al Culture-independent identification of pathogenic bacteria and polymicrobial infections in the genitourinary tract of renal transplant recipients. Journal of clinical microbiology. 2003;41(12):5500–10. Epub 2003/12/10. 10.1128/JCM.41.12.5500-5510.2003 14662931 PMC309025

[29] Chen J, Wright K, Davis JM, Jeraldo P, Marietta EV, Murray J, et al An expansion of rare lineage intestinal microbes characterizes rheumatoid arthritis. Genome medicine. 2016;8(1):43 Epub 2016/04/23. 10.1186/s13073-016-0299-7 27102666 PMC4840970

[30] Funke G, von Graevenitz A, Clarridge JE, Bernard KA. Clinical microbiology of coryneform bacteria. Clinical Microbiology Reviews. 1997;10(1):125–59. PMC172946. 8993861 10.1128/cmr.10.1.125 PMC172946

[31] Morio F, Jean-Pierre H, Dubreuil L, Jumas-Bilak E, Calvet L, Mercier G, et al Antimicrobial susceptibilities and clinical sources of Dialister species. Antimicrob Agents Chemother. 2007;51(12):4498–501. Epub 2007/10/10. 10.1128/AAC.00538-07 17923492 PMC2167981

[32] Tito RY, Cypers H, Joossens M, Varkas G, Van Praet L, Glorieus E, et al Brief Report: Dialister as a Microbial Marker of Disease Activity in Spondyloarthritis. Arthritis & rheumatology (Hoboken, NJ). 2017;69(1):114–21. Epub 2016/07/09. 10.1002/art.39802 . 27390077

