# Supplementary material for: Comparative analysis of the vaginal microbiome of pregnant women with either Trichomonas vaginalis or Chlamydia trachomatis
Source: PLoS One. 2019 Dec 12;14(12):e0225545. doi: 10.1371/journal.pone.0225545 (PMC6907840; doi:10.1371/journal.pone.0225545)
Supplement: S1 File — Table A. Socio-demographic and behavioral characteristics of women selected as controls and women not selected as controls, attending antenatal care at Kilifi County Hospital, Kenya. Figure A. Bar graph showing relative abundance of vaginal bacteria at family level for the three groups. Bar graphs of taxa that are significantly different annotated as *: p<0.05, **: p<0.01, ***: p<0.001 for pair-wise comparisons by t-test for the three groups of women. Figure B. Bacterial α-diversity using Simpson and Shannon indices of women based on Nugent scores. Legend. Intermediate Nugent score; 4–6 (n = 11), Normal Nugent score; (0–3) (n = 42). Figure C. Bar graph showing relative abundance at family level based on Nugent scores. The bar chart display taxa that are significantly different taxa based on an Anova analysis Only taxa that are significantly different with a p-value < 1 are shown A Pair-wise comparisons is then done by t-test and annotated as *: p<0.05, **: p<0.01, ***: p<0.001 Standard error is depicted by error bars. Intermediate Nugent score: 4–6 (n = 11), Normal Nugent score: 0–3 (n = 42). (DOCX) [file pone.0225545.s001.docx]

Table A. Socio-demographic and behavioral characteristics of women selected as controls and women not selected as controls, attending antenatal care at Kilifi County Hospital, Kenya.

| **Characteristic** | **Selected as controls (%)**  **N = 21** | **Not selected as controls (%)**  **N = 251** | **χ2**  **P-value** |
| --- | --- | --- | --- |
| **Age group (Years)** |  | | |
| 18-24 | 28.6 | 36.4 |  |
| ≥ 25 | 71.4 | 63.6 | 0.474 |
| **Religion** |  | | |
| Christian | 66.7 | 72.3 |  |
| Muslim | 9.5 | 15.8 |  |
| Other/None | 23.8 | 11.9 | 0.251 |
| **Education** |  | | |
| None | 19.1 | 17.0 |  |
| Primary | 61.9 | 57.7 |  |
| Secondary/Tertiary | 19.0 | 25.3 | 0.814 |
| **Parity** |  | | |
| 0 | 19.1 | 26.4 |  |
| 1-2 | 42.9 | 36.8 |  |
| 3+ | 38.1 | 36.8 | 0.753 |
| **Gestational age (weeks)** |  | | |
| 14-27 | 66.7 | 60.3 |  |
| ≥ 28 | 33.3 | 39.6 | 0.567 |
| **Number of lifetime sex partners** |  | | |
| ≤ 2 | 95.2 | 88.8 |  |
| ≥3 | 4.8 | 11.2 | 0.362 |

**Figure A. Bar graph showing relative abundance of vaginal bacteria at family level for the three groups**

**
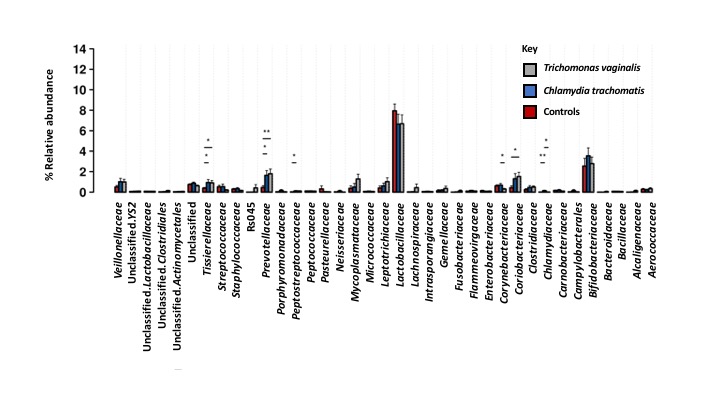
**

**Legend.** Bar graphs of taxa that are significantly different annotated as *: p<0.05, **: p<0.01, ***: p<0.001 for pair-wise comparisons by t-test for the three groups of women.

Figure B. Bacterial α-diversity using Simpson and Shannon indices of women based on Nugent scores.


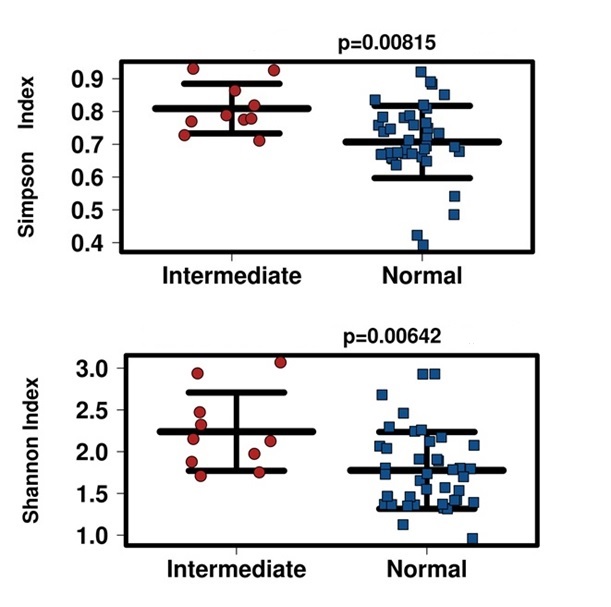


**Legend.** Intermediate Nugent score; 4-6 (n = 11), Normal Nugent score; (0-3) (n = 42).

**Figure C. Bar graph showing relative abundance at family level based on Nugent scores**


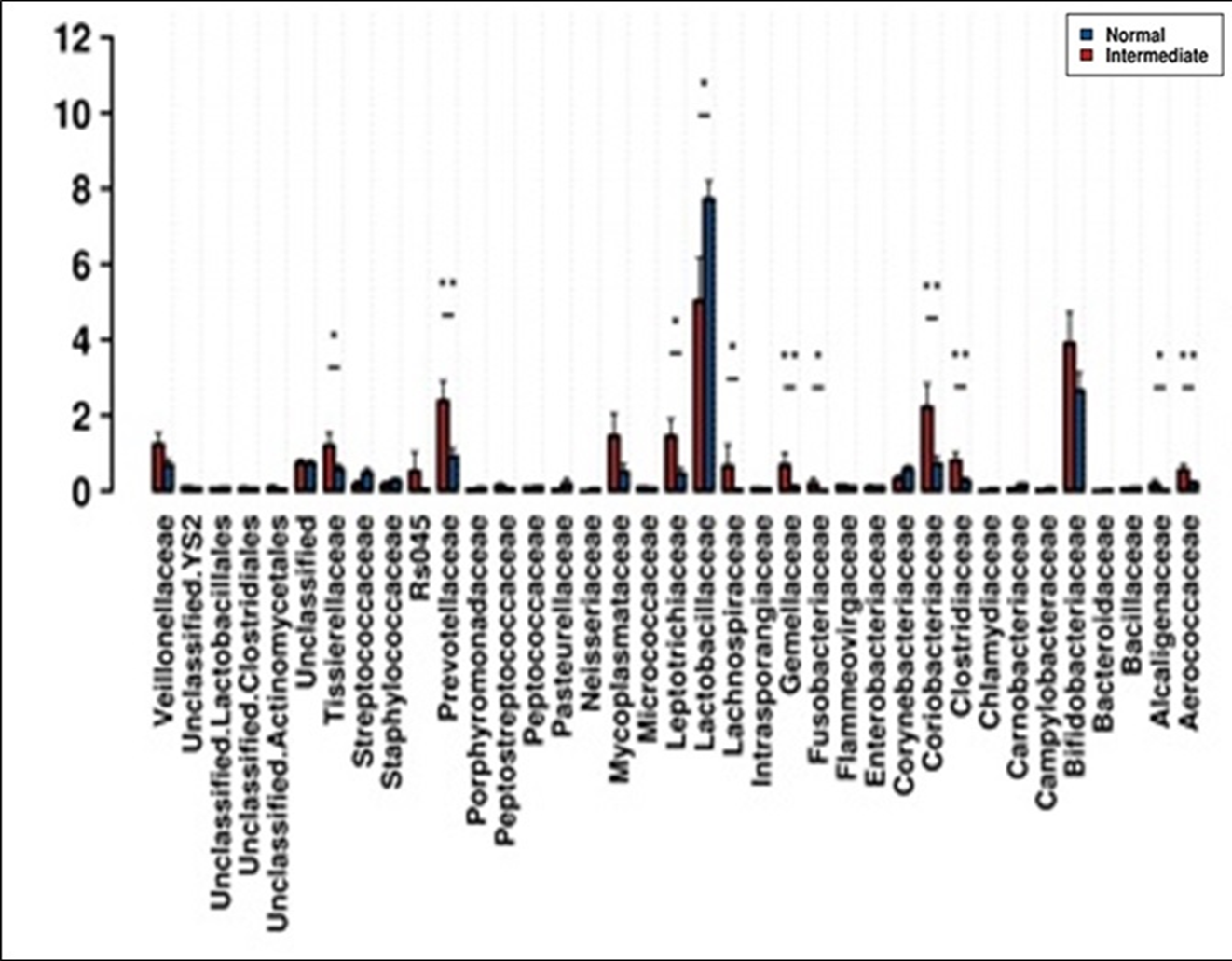


The bar chart display taxa that are significantly different taxa based on an Anova analysis Only taxa that are significantly different with a p-value < 1 are shown A Pair-wise comparisons is then done by t-test and annotated as *: p<0.05, **: p<0.01, ***: p<0.001 Standard error is depicted by error bars. Intermediate Nugent score: 4-6 (n = 11), Normal Nugent score: 0-3 (n = 42).
